# Hyperandrogenism by Liquid Chromatography Tandem Mass Spectrometry in PCOS: Focus on Testosterone and Androstenedione

**DOI:** 10.3390/jcm10010119

**Published:** 2020-12-31

**Authors:** Giorgia Grassi, Elisa Polledri, Silvia Fustinoni, Iacopo Chiodini, Ferruccio Ceriotti, Simona D’Agostino, Francesca Filippi, Edgardo Somigliana, Giovanna Mantovani, Maura Arosio, Valentina Morelli

**Affiliations:** 1 Endocrinology Unit, Fondazione IRCCS Ca’ Granda Ospedale Maggiore Policlinico, 20122 Milan, Italy; giovanna.mantovani@unimi.it (G.M.); maura.arosio@unimi.it (M.A.); valentina.morelli@policlinico.mi.it (V.M.); 2 Department of Clinical Sciences and Community Health, University of Milan, 20122 Milan, Italy; elisa.polledri@unimi.it (E.P.); silvia.fustinoni@unimi.it (S.F.); edgardo.somigliana@unimi.it (E.S.); 3 Laboratory of Toxicology, Foundation IRCCS Ca’ Granda Ospedale Maggiore Policlinico, 20122 Milan, Italy; 4 Department of Medical Biotechnology and Translational Medicine, University of Milan, 20122 Milan, Italy; iacopo.chiodini@unimi.it; 5 IRCCS Istituto Auxologico, Unit for Bone Metabolism Diseases and Diabetes & Lab of Endocrine and Metabolic Research, Italiano, 20149 Milan, Italy; 6 Clinical Laboratory, Fondazione IRCCS Ca’ Granda Ospedale Maggiore Policlinico, 20122 Milan, Italy; ferruccio.ceriotti@policlinico.mi.it (F.C.); simona.dagostino@policlinico.mi.it (S.D.); 7 Infertilty Unit, Fondazione IRCCS Ca’ Granda Ospedale Maggiore Policlinico, 20122 Milan, Italy; francesca.filippi@policlinico.mi.it

**Keywords:** polycystic ovary syndrome, liquid chromatography tandem mass spectrometry, hyperandrogenism, androstenedione

## Abstract

The identification of hyperandrogenism in polycystic ovary syndrome (PCOS) is concerning because of the poor accuracy of the androgen immunoassays (IA) and controversies regarding which androgens should be measured. The aim of our study was to evaluate the impact of the assessment of testosterone (T) and androstenedione (A) by liquid chromatography in tandem with mass spectrometry (LC/MS-MS), in the diagnosis of PCOS. We evaluated 131 patients referred for suspected PCOS. Fourteen patients in total were excluded, some because of other diagnosis (*n* = 7) or incomplete diagnostic workup (*n* = 7). We measured T and A both by IA and LC-MS/MS in the 117 subjects included. We calculated free T (fT) by the Vermeulen formula and recorded clinical and metabolic data. 73 healthy females served as controls to derive immunoassays (IA) and LC-MS/MS reference intervals for T, fT and A. PCOS was confirmed in 90 subjects by IA and in 93 (+3.3%) by LC-MS/MS. The prevalence of biochemical hyperandrogenism in PCOS by LC-MS/MS increased from 81.7% to 89.2% if A was also considered. The most frequently elevated androgens were fT (73.1%) and A (64.5%) and they had similar levels of accuracy in differentiating PCOS and controls (0.34 ng/dL, Sn 91% Sp 89%; 1.16 ng/mL, Sn 91% Sp 88%, respectively). Free testosterone correlated with body mass index (BMI), homeostatic model assessment (HOMA)-index, glycated hemoglobin (HbA1c), and sex-binding globulin (SHBG). The results confirm that LC-MS/MS is slightly more sensitive than IA in the diagnosis of PCOS with LC-MS/MS detecting higher levels of fT and A. Moreover, assessment of fT and A by LC-MS/MS had a similar level of accuracy in discriminating between PCOs and control subjects. Lastly, fT by LC-MS/MS correlates with adverse metabolic parameters.

## 1. Introduction

Polycystic ovary syndrome (PCOS) is a common endocrinological disorder, with a prevalence as high as 9–18% in females of reproductive age, and it is associated with reproductive and metabolic dysfunctions [1,2]. The diagnosis is based on the association of two conditions from the following: oligoanovulation (OA), micropolycystic ovarian morphology (mPCO), and hyperandrogenism (Ha) (which can be either defined by clinical (hirsutism, acne, or alopecia) or biochemical (high androgens concentrations) findings) [3,4]. However, the assessment of clinical hyperandrogenism is challenging because its quantification relies on visual scores that are ladened with inter-observer variability [5]. Although current guidelines recommend the use of reliable steroid measurement methods, this is not always feasible in clinical practice because of their inaccessibility and high costs. In spite of this, immunoassays, which lack the sensitivity and accuracy in measuring androgens in woman, remain the most utilized technique [6,7]. Furthermore, there is no general agreement about which androgen should be measured, as several hormonal sources and enzymatic pathways (including the 11-oxygenated C19 steroid pathway) have been implicated in the pathogenesis of PCOS [8,9]. Current guidelines recommend the measurement of total testosterone (T) and free testosterone (fT) initially, and androstenedione (A) is added when normal T and fT concentrations are obtained [4]. It has been reported that A represents an essential marker of PCOS, providing critical information about metabolic risk. Moreover, some authors argue that a multi-steroid profile could be more sensitive in detecting hyperandrogenism in these patients [10,11,12]. It is likely that the use of liquid chromatography in tandem with mass spectrometry (LC-MS/MS), the gold standard for steroids measurements, allowing simultaneous measurement of several androgens, may improve the diagnosis and phenotyping of PCOS patients [13]. However, this technique has had limited application in clinical practice because of its complexity, and its difficulty in preparing quality control samples, which increased with the number of analytes. Recently, a commercial product, specifically developed for the measurement of steroid panels by LC-MS/MS, has become commercially available.

The aim of our study was to assess the impact of the use of LC-MS/MS in the diagnosis of PCOS in a group of patients referred to our center. Moreover, our study determined which was the most accurate androgen to discriminate between PCOS and controls. We also correlated androgen concentrations and metabolic parameters.

## 2. Experimental Section

### 2.1. Subjects

We evaluated 131 female patients of reproductive age referred to the Endocrinology Unit of the IRCCS Fondazione Ca’ Granda Ospedale Maggiore Policlinico for suspected PCOS over a period of 2 years. We included subjects with the following characteristics: age ≥ 18 years, clinical hyperandrogenism (hirsutism, alopecia, or acne) and/or menstrual irregularities, not interfering drugs (i.e., estroprogestinics, glucocorticoids), and not secondary forms of PCOS (hyperprolactinemia, non-classic congenital adrenal hyperplasia, hypothyroidism, hypercortisolism, acromegalia, etc.). We excluded seven patients because they did not complete the diagnostic framework and another seven patients because they received other diagnosis or had secondary forms of PCOS (non-classic congenital adrenal hyperplasia, *n* = 3; hypogonadotropic hypogonadism, *n* = 4). Of the 131 patients referred to the Endocrinology unit, 117 were included in our study.

73 lean Caucasian women of reproductive age (age 33.5 ± 3.6 years, BMI 20.9 ± 3.6 kg/m^2^) with regular menses, no ultrasonographic findings of mPCO, and no clinical signs of hyperandrogenism served as controls to derive immunoassay (IA) and LC-MS/MS reference intervals for T (0.05–0.45 and 0.11–0.34 ng/mL), fT (0.05–0.57 and 0.11–0.41 ng/dL), and A (0.1–3.0 and 0.4–1.6 ng/mL; respectively.

The study protocol was approved by the local Ethics Committee, and written informed consent was obtained from all the participants.

### 2.2. Methods

Each subject underwent a medical examination and blood sampling. We recorded height, weight, waist circumference, arterial pressure, BMI, and the Ferryman Gallwey Score. Blood samples were obtained at a fasting state in the follicular phase in subjects with regular menses or mild oligomenorrhoea, and after bleeding induced by progestin withdrawal in subjects with severe oligomenorrhea or amenorrhea. We measured luteinizing hormone (LH), follicle-stimulating hormone (FSH), glucose, insulin, total cholesterol, HDL-c, triglycerides, albumin, sex-binding globulin (SHBG), T, A, and dehydroepiandrosterone sulphate (DHEAS). Sex-hormone binding globulin (SHBG) was measured by Immulite 2000 (Siemens Healthineers, Milano, Italy). We calculated fT using the Vermeulen’s formula and the free-androgen index (FAI) with the formula Tx100/SHBG [14]. The diagnosis of PCOS was confirmed according to more recent guidelines [9]. Oligo-anovulation was defined as the presence of irregular menstrual cycles (amenorrhoea, oligomenorrhoea: cycle duration >35 days or <8 menstrual cycles per year, polymenorrhoea: cycle duration < 21 days) or anovulation (progesterone concentrations <2 ng/mL or absence of corpus luteus in the luteal phase) [4]. Polycystic ovarian morphology was evaluated through an endovaginal ultrasound or, when not feasible, transabdominal examination using a transducer frequency ≥8 MHz. We defined a polycystic ovary morphology (mPCO) if the antral follicular count (AFC) was ≥20 or the ovarian volume was ≥10 cm^3^ [4]. Clinical hyperandrogenism was defined by the finding of alopecia, acne, or hirsutism (mFG score > 5), whereas biochemical hyperandrogenism was defined by high T, fT, or A concentrations. Secondary causes of PCOs (hypercortisolism, non-classic congenital adrenal hyperplasia, hyperprolactinemia, acromegaly, androgen-secreting tumors, drugs, and thyroid dysfunction) were excluded by clinical and biochemical assessment. The enrolled subjects were classified as non-PCOS or PCOS initially, based on T, fT, and A levels assessed by IA. The impact of LC-MS/MS on the diagnosis of PCOS was then evaluated by measuring T, fT, and A levels using LC-MS/MS.

### 2.3. Assays

All hormone measurements were performed in the same laboratory.

Androgens were measured both by IA (Cobas Elecsys Testosterone II, CV 5.5% LOQ 0.12 µg/L; Immulite 2000 Androstenedione CV 10%, LOQ 0.3 µg/L) and by the IVD-MS steroids in serum kit (MassChrom, Steroids in Serum/Plasma, Chromsystems, Gräfelfing, Germany). These tests were followed by an analysis with LC-MS/MS (CV inter-assay: 6% A and 5% T; CV intra-assay: 2% A and 2% T; LOQ A: 0.02 µg/L and T 0.005 µg/L). Chromatographic separation and mass spectrometric detection were performed with a high-performance liquid chromatography (Agilent, Torino, Italy) interfaced with a Sciex 5500 QTRAP mass spectrometer (Sciex, Milano, Italy). Samples were prepared according to the manufacturer’s instructions. Briefly, 500 µL of each sample, calibrators, or quality control (QC) serum were placed in a previously equilibrated SPE sample plates, to which 50 µL of a deuterated internal standard mix solution as well as 450 µL extraction buffer were added. This mixed sample was then vortexed and centrifuged. The supernatant was evaporated under nitrogen to dryness and reconstituted with 100 µL of reconstitution buffer, and a 40 µL aliquot was injected into the LC system equipped with an analytical column (operating at 32 °C) for peak separation. Mobile phases A and B (provided with the kit) were used for steroid elution. For the quantification of the analytes, a blank calibrator matrix and six multilevel serum calibrators, provided with the kit, were used. Three certified QCs serum, provided with the kit, were used to assess within- and between-run precision and accuracy. During routine analyses, calibration curves and QCs were run within each set of unknown samples. A typical analytical sequence consisted of a calibration curve, followed by unknown samples, with QC samples repeated every ten unknown samples, and followed by a second calibration curve.

### 2.4. Statistical Analysis

Statistical analysis was performed using the SPSS version 25.0 statistical package (SPSS Inc., Chicago, IL, USA), and MedCalc statistical software for Windows (Ostend, Belgium). A comparison of continuous variables was performed using the t-test or Mann–Whitney test, as appropriate. The normality of distribution was checked by the Kolmogorov–Smirnov test. Variables were expressed as mean ± standard deviation if normally distributed, and as median (interquartile range) if not normally distributed. The comparisons were performed using a one-way Student t-test or Mann–Whitney U-test, respectively, as appropriate. A p-value less than 0.05 was considered significant. Bivariate associations were tested by Pearson product-moment (r) or Spearman’s rank correlation (rs), as appropriate. The accuracy of T, fT, FAI, and A in discriminating between PCOS patients and controls was estimated using the area under the curve receiver operating characteristics (ROC) curves.

## 3. Results

In all the 117 subjects evaluated, androgen concentrations measured by LC-MS/MS tended to be lower than by IA, although statistically significant differences were observed only for A (0.42 interquartile range, IQR 0.22 vs. 0.38 IQR 0.23 ng/mL, *p* = 0.221; 0.64 IQR 0.46 vs. 0.58 IQR 0.42 ng/dL, *p* = 0.369; 2.5 IQR 1.9 vs. 1.8 IQR 0.6, *p* = 0.001; respectively, for T, fT, and A by IA and LC-MS/MS). PCOS was diagnosed in 90/117 patients by routine IA and in 93/117 patients (+3.3%) by LC-MS/MS.

With regards to the diagnosis by LC-MS/MS, we compared clinical, metabolic, and biochemical parameters between non-PCOS and PCOS subjects (Table 1). We found that PCOS patients had significantly higher ovarian volume, NFPO, and LH concentrations and were more frequently oligomenorroeic than non-PCOS subjects. Although BMI and waist were comparable between the two groups, insulin concentrations and HOMA-index were significantly higher in PCOS patients, and HDL and SHBG concentrations were lower. The concentrations of T, fT, and A tended to be higher in PCOS patients both by LC-MS/MS and IA; however, we found statistically significant differences only in FAI by both methods. We categorized PCOS patients into Rotterdam phenotypes, according to the various combinations of oligoamenorrhea (OA), clinical and/or biochemical hyperandrogenism (Ha), and polycystic ovaries (mPCO). The prevalence of the different phenotypes in PCOS patients was *n* = 40 (43.2%) complete (OA+mPCO+Ha) phenotype, *n* = 39 (42%) classic (OA+Ha) phenotype, *n* = 13 (13.7%) ovulatory (Ha+mPCO) phenotype, and *n* = 1 (1.1%) normoandrogenic (OA+mPCO) phenotype. The comparison among the Rotterdam PCOS phenotypes revealed that the prevalence of subjects with high androstenedione measured with LC-MS/MS (hA-TM) was significantly higher in the classic phenotype versus both complete (77.1 vs. 55.6%, *p* = 0.050) and ovulatory phenotype groups (77.1 vs. 45.5%, *p* = 0.046). Moreover, they did not differ in clinical, metabolic, and biochemical parameters or other steroids concentrations (data not shown).

**Figure 1:**
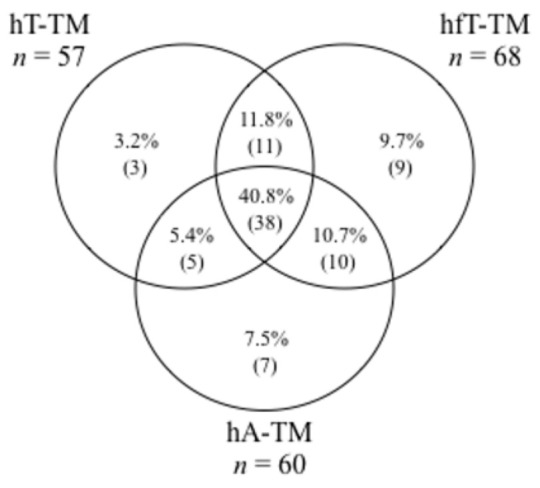
Distribution of the patterns of biochemical hyperandrogenism based by LC-MS/MS androgens quantification in PCOS patients. hT-TM: high testosterone by LC-MS/MS. hfT-TM: high free testosterone by LC-MS/MS. hA-TM: high androstenedione by LC-MS/MS.

**Figure 2:**
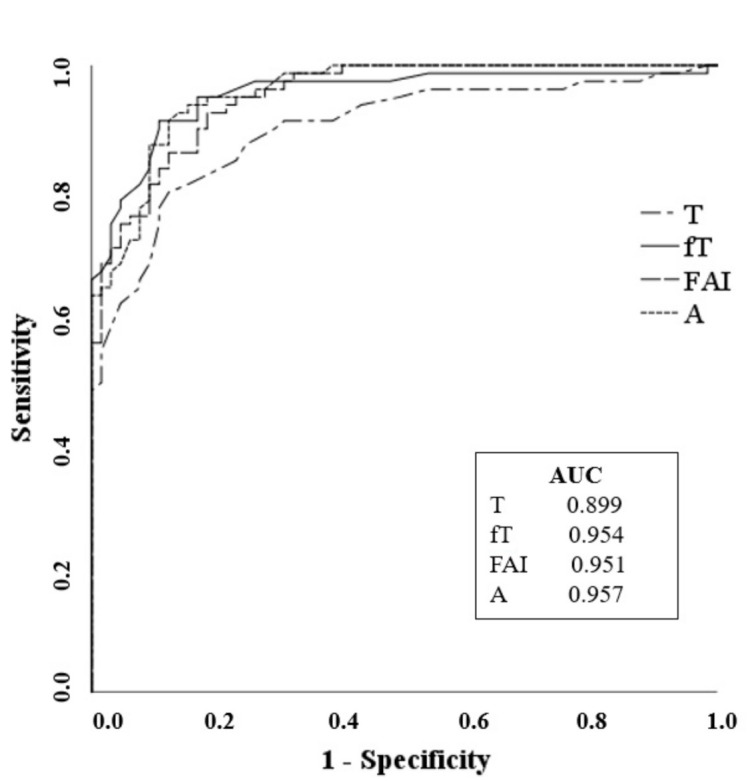
The receiver operating characteristics (ROC) curve analyses of T, fT, free androgen index (FAI), and A measured by LC-MS/MS in discriminating PCOS patients from controls.

Biochemical hyperandrogenism (high T and/or fT and/or A by LC-MS/MS) was present in 83 (89.2%) PCOS patients. The distribution of the different biochemical hyperandrogenism patterns in PCOS patients is shown in Figure 1. The results demonstrated that fT was the most frequently elevated androgen (68/93, 73.1%), followed by A (60/93, 64.5%). Moreover, 40.8% (38/93) of patients presented simultaneous high levels of T, fT, and A. The ROC analysis showed a high and comparable accuracy for A (cut off 1.16 ng/mL, Sn 91% Sp 88%) and fT (cut off 0.34 ng/dL, Sn 91% Sp 89%) in differentiating PCOS patients from controls, while FAI (cut off 1.67, Sn 90% Sp 83%) and T (cut off 0.24 ng/mL, Sn 87% Sp 75%) had poorer accuracy (Figure 2).

In PCOS patients, neither A nor T measured by LC-MS/MS correlated with any clinical or metabolic parameters. However, we found a significant correlation between LC-MS/MS fT concentrations and BMI (r = 0.346, *p* = 0.002), HOMA-index (0.250, *p* = 0.042), HbA1c (r = 0.337, *p* = 0.025), and SHGB (r = −0.353, *p* = 0.002). In addition, FAI calculated based on LC-MS/MS data was significantly correlated with BMI (r = 0.398, *p* = 0.001), HOMA-index (r = 0.289, *p* = 0.016), waist (r = 0.408, *p* = 0.001), and SHBG (r = −0.353, *p* = 0.002). Moreover, SHBG was the biochemical marker that best correlated with the following metabolic parameters: BMI (r = −0.531, *p* = 0.001), waist (r = −0.502, *p* = 0.001), HOMA-index (r = −0.468, *p* = 0.001), HbA1c (r = −0.300, *p* = 0.046), and hirsutism severity (r = −0.404, *p* = 0.001).

## 4. Discussion

In our cohort, the use of the LC-MS/MS technique led to a slightly increased number of PCOS diagnosis (+3.3%), compared with IA. PCOS patients exhibited a higher HOMA-index and lower levels of HDL-C and SHBG than non-PCOS subjects. Biochemical hyperandrogenism (high T and/or fT and/or A by LC-MS/MS) was identified in nearly 90% of PCOS. Free testosterone concentrations correlated with metabolically adverse parameters, and it was the most frequently elevated androgen in our cohort, followed by A. The ROC curve analysis demonstrated that fT and A concentrations were the best in discriminating between PCOS patients and controls.

The identification of hyperandrogenism is the cornerstone for the assessment of PCOS. However, its definition has always been concerning because of the poor accuracy shown by routine androgen assays and the controversies regarding which androgen should be assessed. According to current guidelines, androgens should preferably be measured using highly accurate methods such as LC-MS/MS. The LC- MS/MS method allows for the simultaneous measurement of several steroids, which ensures rapid analysis times and improves specificity compared with IA. However, because it is not easily accessible, its application in the routine clinical setting has not been widespread. Moreover, the interpretation of the results has been complicated by the complexity and difficulty in preparing calibrators and quality control samples and by the lack of unambiguous cut-offs. Some laboratories are now using commercially available kits specifically developed for the measurement of steroid panels in LC-MS/MS assays. Standard reference intervals could be developed from shared data, resulting in the major benefit of harmonising results interpretation in different centres. It has been suggested that the application of LC-MS/MS could improve the assessment of hyperandrogenism and the diagnostic workup of PCOS. However, a recent meta-analysis on this subject concluded that there are still not enough studies available to draw definitive conclusions [15]. In line with other authors who have reported a minimal improvement in the diagnosis of PCOS in our cohort the use of the LC-MS/MS commercially available kit for measuring T and A led to a slight increase (+3.3%) in the number of PCOS patients identified compared with IA [16]. It is likely that the measurement of the 11-oxygenated androgens (11β-hydroxyandrostenedione, 11-ketotestosterone and 11-keto-5α-dihydrotestosterone) by LC-MS/MS could lead to the identification of a higher proportion of PCOS patients, although, to the best of our knowledge, there are currently no commercially available internal standards that could limit their use in the clinical setting [8].

We have confirmed previous reports that lower hormonal concentrations are generally found using LC-MS/MS when compared to IA [17,18]. We also obtained from a control group (*n* = 73) sex- and age-specific reference ranges for A (0.4–1.6 ng/mL) and T (0.11–0.34 ng/mL). Moreover, we calculated fT (0.11–0.41 ng/dL) by the MassChrome LC-MS/MS kit, which, to the best of our knowledge, has not previously been reported in the literature. Our results are similar to other studies where other kits were used [19].

When measuring T and/or A by LC-MS/MS and/or fT calculated by the Vermeulen formula, we found that a biochemical hyperandrogenism was present in 89.1% of PCOS patients. Similar results were found by other authors based on A in combination with T or FAI [7,10,11].

In our patients, fT was the most frequently elevated androgen, followed by A, and 40.8% of patients presented with concomitantly high levels of T, fT, and A, supporting previous findings [6,10,11]. The ROC curve analyses showed that, by LC-MS/MS analysis, the androgen A could discriminate between PCOS patients and controls similarly to fT and more accurately than T or FAI. The same results were reported by Pasquali et al. [10]. Our data supports the suggestion that steroid profiling that includes the androgen A is helpful in the correct identification of hyperandrogenic PCOS patients, and that A is as accurate as fT in differentiating PCOS patients and controls. It is a critical finding since fT determination or its cut-off are not always available.

In our study, we found that fT concentrations correlated with metabolically adverse parameters (BMI, HOMA-index, and HbA1c), whereas there was no correlation between A and metabolic aspects. This is in contrast with the findings of O’Reilly et al. that described a significant association between high A concentrations and an adverse metabolic phenotype, but confirms the results in the metanalyses by Amiri et al. [11,12,13,14,15].

Our study had some limitations that could have affected the results observed. First, our PCOS population was unique, probably due to the new ultrasound criteria for mPCO definition; consequently, very few subjects with the Rotterdam OA+mPCO phenotype were found. Secondly, overall, our patients were more lean and with a more favourable metabolic profile.

Our findings are consistent with previous studies, although other studies could evaluate the proportion of “false positives” by immunoassays compared with LC-MS/MS. However, no study could estimate the amount of “false negatives” in a cohort of patients with suspected PCOS. In addition, most articles on this topic were published before the release of the most recent guidelines on PCOS management. We used these current guidelines to diagnose PCOS in our study resulting in a more precise screening of our patient cohort [9]. We also derived LC-MS/MS reference intervals from a larger control group than other studies [11,12,13,14,15,16]. Furthermore, we used a commercially available multisteroid LC-MS/MS kit for androgens measurement and obtained concentrations comparable to previous studies in which domestic kits were used. This finding confirms the reproducibility of more advanced techniques that allow for simultaneous measurement of several androgens in a single sample [6,7,8].

In conclusion, we have provided evidence that, in the diagnosis of PCOS, quantification of androgens based on the LC-MS/MS technique is slightly more sensitive than those based on IA assays. The measurement of A by LC-MS/MS should be routinely performed in suspected PCOS patients, especially if fT is not available, as it can discriminate from controls more accurately than T. A is also useful to correctly identify hyperandrogenic PCOS subjects, although its role in metabolic risk assessment remains unclear.

## Figures and Tables

**Table 1:** Clinical and biochemical characteristics of patients diagnosed as polycystic ovary syndrome (PCOS) or non-PCOS considering testosterone (T), free testosterone (fT), and androstenedione (A) measurement by liquid chromatography in tandem with mass spectrometry (LC-MS/MS).

	All Patients (*n* = 117)	Non-PCOs (*n* = 24)	PCOs (*n* = 93)	*p*
Age (years)	23.7 ± 0.6 (18–39)	23.9 ± 5.8 (18–36)	23.7 ± 6.3 (18–39)	0.921
BMI (kg/m^2^)	23.2 (7.4)	22.6 (5.4)	23.5 (8.1)	0.858
Waist (cm)	80 ± 15.8 (60–126)	74.3 ± 7.4 (66–88)	81.6 ± 17.1 (60–126)	0.179
Ovarian Volume (mL)	11.5 ± 0.6 (4–27.5)	7.3 ± 2.9 (4.3–12.4)	11.9 ± 0.7 (3.5–27.2)	0.004
AFC	17 ± 7 (3–33)	11 ± 6 (3–20)	19 ± 6 (6–33)	0.001
mFG	10 ± 8 (0–27)	7 ± 6 (0–18)	10 ± 7 (0–33)	0.119
HOMA index	1.8 (1.9)	1.4 (1.1)	1.8 (2)	0.032
PaS (mmHg)	111 ± 11 (90–145)	115 ± 12 (90–135)	113 ± 13 (85–145)	0.594
PaD (mmHg)	71 ± 10 (55–100)	75 ± 10 (60–100)	73 ± 9 (55–95)	0.354
Glucose (mg/dL)	84 ± 6 (72–97)	83 ± 6 (66–93)	84 ± 10 (63–145)	0.730
Insulin (mIU/L)	8.7 (8.4)	7.2 (6.7)	9.1 (8.2)	0.024
HbA1c (mmol/mol)	32 (3)	33 (3)	32 (4)	0.455
Total cholesterol (mg/dL)	155 (31)	165 (38)	155 (31)	0.089
HDL-C (mg/dL)	56 ± 15 (31–91)	63 ± 10 (49–91)	55 ± 15 (30–100)	0.020
LDL-C (mg/dL)	85 ± 29 (28–143)	90 ± 20 (50–130)	84 ± 25 (28–143)	0.350
Triglycerides (mg/dL)	64 (29)	56 (32)	66 (42)	0.149
PRL (mIU/L)	12.7 (6.3)	13.2 (8.3)	12.6 (6.4)	0.245
LH (mIU/L)	6.2 (5.5)	4.5 (2.9)	6.3 (5.6)	0.015
FSH (mIU/L)	5.5 (1.7)	6.0 (1.9)	5.4 (1.7)	0.204
17bE (mg/dL)	33.6 (20.8)	34.4 (19.7)	33.6 (22)	0.494
SHBG (nmol/L)	43.3 ± 23.3 (12–114)	51.5 ± 21.4 (22–104)	41.2 ± 23.4 (11.6–114)	0.071
T-IA (ng/mL)	0.42 (0.22)	0.39 (0.19)	0.43 (0.23)	0.203
T-TM (ng/mL)	0.38 (0.23)	0.35 (0.2)	0.40 (0.22)	0.116
fT-IA (ng/dL)	0.64 (0.46)	0.57 (0.31)	0.68 (0.48)	0.059
fT-TM (ng/dL)	0.58 (0.42)	0.47 (0.35)	0.58 (0.45)	0.099
A-IA (ng/mL)	2.5 (1.9)	2.4 (1.0)	2.7 (2.0)	0.18
A-TM (ng/mL)	1.8 ± 0.6 (0.9–3.8)	1.6 ± 0.6 (0.9–3.5)	1.9 ± 0.6 (0.9–3.8)	0.068
FAI-IA	0.4 (0.4)	3.1 (2.3)	3.8 (4.6)	0.002
FAI-TM	3.3 (0.5)	2.6 (2.0)	3.5 (3.6)	0.012
DHEAS-IA (ng/mL)	3027 ± 1409 (250–8169)	3159 ± 1365 (999–6055)	2991 ± 1426 (250–8169)	0.607

Data are expressed as mean ± standard-deviation, median, and interquartile-range in brackets. BMI: body mass index. OA: oligomenorrhea. mPCO: polycistic ovaries. AFC: antral follicular count. mFG: modified Ferriman Gallway score. PaS: sistolic arterial pressure. PaD: diastolic arterial pressure. T: total testosterone. fT: calculated free testosterone. A: androstenedione. PRL: prolactin. 17bE: 17 beta oestradiol. SHBG: sex hormones binding globulin. IA: immuno assay. FAI: free androgen index. TM: LC-MS/MS.

## Data Availability

The data presented in this study are available on request from the corresponding author. The data are not publicly available due to privacy.
